# Retrospect of Uterine Pathology and Therapeutics in the United States

**Published:** 1871-11

**Authors:** 


*Retrospect of Uterine Pathology and Therapeutics in the United States.* By Henry Miller, M.D.

This paper treats specially of intra-uterine medication in chronic internal metritis. The author claims to have been the first to introduce in this country intra-uterine medication. He gives a brief notice of medical books and journals which contained notices of endometritis and its medication since 1855.

